# First description of sarcoptic mange in an Iberian hare (*Lepus granatensis*)

**DOI:** 10.1016/j.crpvbd.2021.100021

**Published:** 2021-03-24

**Authors:** Jesús Cardells, Victor Lizana, Alba Martí-Marco, Santiago Lavín, Roser Velarde, Luca Rossi, Barbara Moroni

**Affiliations:** a Servicio de Análisis, Investigación, Gestión de Animales Silvestres (SAIGAS), Facultad de Veterinaria, Universidad Cardenal Herrera-CEU, CEU Universities Alfara del Patriarca, Valencia, Spain; b Wildlife Ecology & Health Group (WE&H), Universitat Autònoma de Barcelona (UAB), Bellaterra, Barcelona, Spain; c Dipartimento di Scienze Veterinarie, Università degli Studi di Torino, Largo Paolo Braccini 2, 10095, Grugliasco, Italy

**Keywords:** *Sarcoptes scabiei*, Iberian hare, Sarcoptic mange, Wildlife, Molecular epidemiology, Microsatellite

## Abstract

The Iberian hare (*Lepus granatensis*) is a popular small game species in the Iberian Peninsula, and it has never been reported to be affected by sarcoptic mange. An adult female Iberian hare with overt skin lesions on forelimbs and ventral thorax, suggestive of sarcoptic mange, was culled in Quart de les Valls municipality in the Valencian Community, Spain, in 2019. Skin scrapings were digested in 10% KOH solutions to confirm the presence of mites. Ten *Sarcoptes* microsatellite markers were used to characterize the genetic structure of mites obtained from the hare, and from sympatric and allopatric wild rabbits (*Oryctolagus cuniculus*) and red foxes (*Vulpes vulpes*). A total of 56 alleles were counted across the 10 microsatellite loci. Six private alleles were found at four loci (Sarms 33, 38, 41, 45). The multivariate analysis characterized three main clusters, corresponding to mites collected on foxes originating from Catalonia, foxes from Valencia and the hare plus wild rabbits. To our knowledge, this is the first reported case of sarcoptic mange in the Iberian hare. The origin was molecularly traced back to contacts with endemically infected wild rabbits. We encourage further investigations on cross-transmissibility of *S. scabiei* between wild rabbits and the diverse representatives of *Lepus* spp.

## Highlights

• Crusted scabies in an Iberian hare was detected for the first time in Valencia, Spain in 2019.
• Wild rabbits might be a natural source of infection of *S. scabiei* for the Iberian hare, rather than sympatric predators.
• Microsatellite markers are efficient epidemiological tools for tracing the source of *Sarcoptes scabiei* infection.

## Graphical abstract

**Figure:**
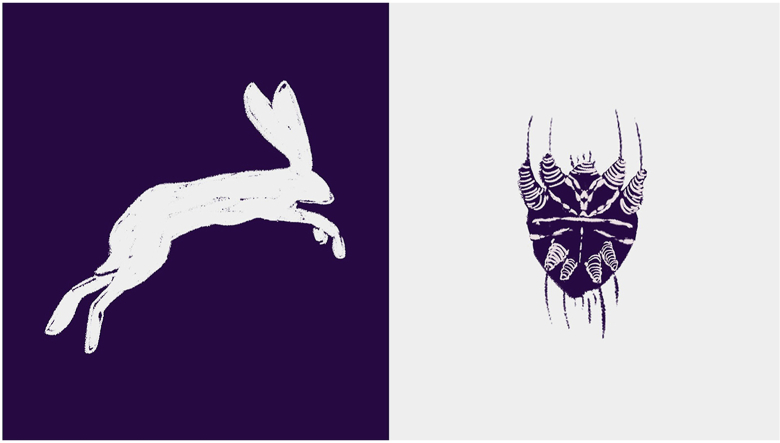

## 1 Introduction

Sarcoptic mange caused by the burrowing mite, *Sarcoptes scabiei*, is a skin disease affecting humans, domestic and wild mammals worldwide (Bornstein et al., 2001; Pence & Ueckermann, 2002; Walton et al., 2004). Mild infection in animals is characterized by pruritic papules, erythema, scales and alopecia, whereas the main sign in chronic cases is skin thickening due to hyperkeratosis and/or exudative crusts formation (Rahman et al., 2010). In Spain, sarcoptic mange has been reported in several game species including the Iberian ibex (*Capra pyrenaica*) (León-Vizcaíno et al., 1999), Southern chamois (*Rupricapra pyrenaica parva*) (Fernández-Morán et al., 1997), roe deer (*Capreolus capreolus*) (Oleaga et al., 2008a), red deer (*Cervus elaphus*) (Oleaga et al., 2008b), wild boar (*Sus scrofa*) (Haas et al., 2018), red fox (*Vulpes vulpes*) (Gortázar et al., 1998), European wild rabbit (*Oryctolagus cuniculus*) (Millán, 2010), and the introduced European mouflon (*Ovis aries musimon*), fallow deer (*Cervus dama*) and Barbary sheep (*Ammotragus lervia*) (Iacopelli et al., 2020; Moroni et al., 2021).

In naïve wildlife populations, infection with *S. scabiei* usually results into high morbidity and mortality (Rossi et al., 2019). In the Cazorla National Park, southern Spain, the infection spread in few months to over 70% of Iberian ibex, resulting in the death of 95% of the clinically affected individuals (Fandos, 1991; León-Vizcaíno et al., 1999). A similarly drastic population decline was observed in several carnivores in Scandinavia, where *S. scabiei* was introduced in the late 1970s (Holt & Berg, 1990; Mörner, 1992). Sarcoptic mange can affect the dynamics of exposed populations, by increasing natural mortality rates (Uraguchi et al., 2014), and altering the spatial behavior of animals (Potts et al., 2013).

The Iberian hare (*Lepus granatensis*) is native to the Iberian Peninsula. The species is widely distributed in agricultural areas and open fields at altitudes ranging between sea level and 1,200 m above the sea level (Duarte, 2000). In northern Spain it may be sympatric with the European brown hare (*Lepus europaeus*) and the vulnerable Broom hare (*Lepus castroviejoi*) (Carro & Soriguer, 2007; Purroy, 2017). The Iberian hare is a popular small game species, with more than one million individuals hunted every year (Vargas, 2002). *Lepus granatensis* is apparently resistant to the European brown hare syndrome, a deadly viral disease in European brown hares and Mountain hares (*Lepus timidus*) (Gavier-Widén & Mörner, 1991; Lopes et al., 2014). However, a new strain of Myxoma virus, previously restricted to rabbits, is causing high mortality in Iberian hares since summer 2018 (Águeda-Pinto et al., 2019; Dalton et al., 2019; García-Bocanegra et al., 2019). The immunosuppression associated to Myxoma virus infection (Jeklova et al., 2008) could represent an open gap to other previously undetected diseases.

The aim of this study was to report the first case of sarcoptic mange in the Iberian hare, and to describe the molecular profile of the *Sarcoptes* mites collected on the new host.

## 2 Materials and methods

During a surveillance campaign for the detection of myxomatosis in hares in the Valencia Community, Spain, an adult female Iberian hare was found dead with overt skin lesions compatible with sarcoptic mange in the Quart de les Valls municipality (39°44′27.96″N, 0°16′17.04″W), Valencian Community, Spain.

The necropsy was carried out at the Veterinary Faculty of the CEU Cardenal Herrera University. Skin samples from forelimbs and ventral thorax were taken and digested in a 10% KOH solution at 37°C overnight for the specimen isolation. Samples were then centrifuged at 3,500 rpm for 5 min, and the supernatant was removed and floated with a saturated sucrose solution for 5 min (Bornstein et al., 2001). To collect individual specimens for molecular studies, crusts were scratched under a stereomicroscope (Leica DM750) in an aqueous medium (Soglia et al., 2009) and delivered to the Veterinary Faculty of Turin (University of Turin, Italy).

DNA extraction was performed from individual mites following the HotSHOT Plus Thermal SHOCK technique (Alasaad et al., 2008). Then, a 10 × multiplex PCR was carried out using 10 validated primers to target *S. scabiei* mites (Sarms 33, 34, 35, 36, 37, 38, 40, 41, 44, 45) (Soglia et al., 2007; Alasaad et al., 2008). Capillary electrophoresis was performed with an ABI PRISM 310 Genetic Analyzer, and the software GeneMapper 4.0 (Applied Biosystems, Foster City, CA, USA) was applied to visualize the microsatellite peaks.

The genetic profile of mites isolated from the hare of this study was compared to those of mites previously collected from wild rabbits and red foxes in Spain.

Population genetics analyses were carried out using Bayesian clustering approach with the software STRUCTURE 2.3.4 (Pritchard et al., 2000), while a multivariate principal components analysis (PCA) with microsatellite marker data as variables was performed with R 4.0 using the package *ade 4.0* (Dray & Dufour, 2007).

Assessments of observed (Ho) and expected heterozygosity (He), allelic richness (R) and the Hardy-Weinberg Equilibrium (HWE) analysis, were carried out with software R 4.0 using the package *Adegenet 2.1.3* (Jombart, 2008).

For the Bayesian analysis, admixture model was selected, “burn-in” and run lengths of Markov chains were 10,000 and 100,000, respectively, and 10 independent runs for each K (for *K* = 1–20) were run. The estimation of clusters was performed using the DK method of Evanno. Individual mites were then associated to the correspondent inferred cluster (Earl & vonHoldt, 2012).

## 3 Results

**Fig. 1:**
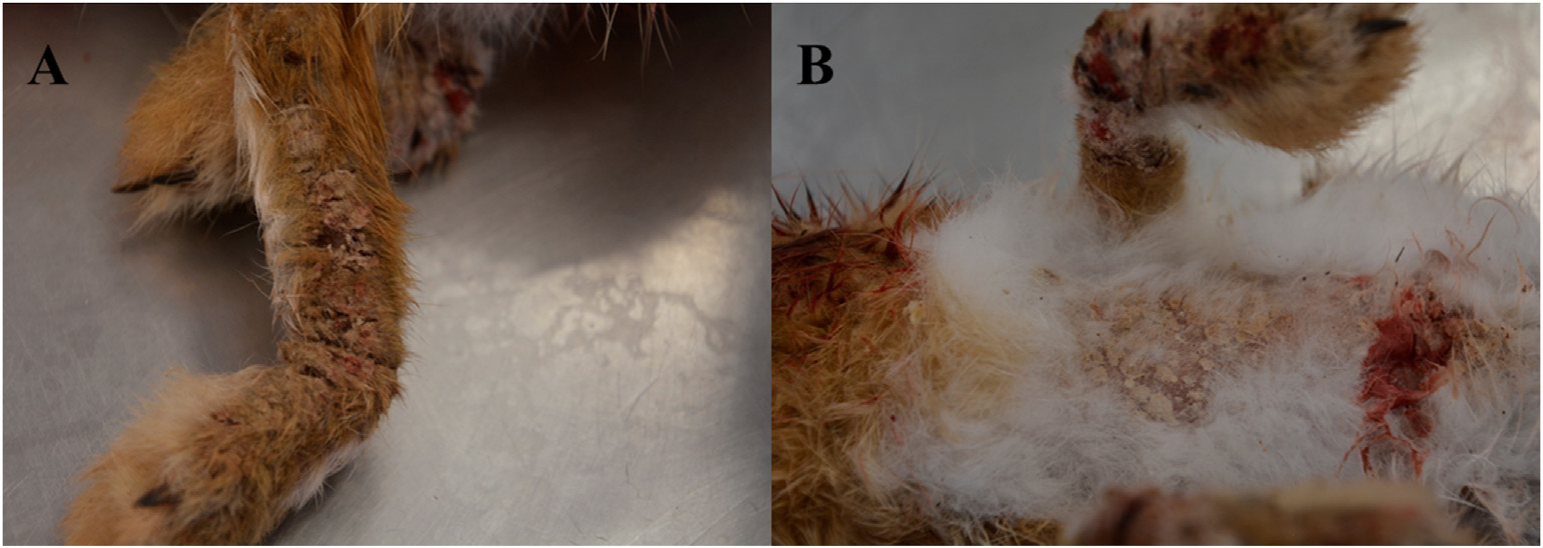
**A** Skin lesions present on the forelimb of the Iberian hare. **B** Skin lesions present on the ventral thorax of the Iberian hare

At the gross examination of the carcass, extended hyperkeratotic skin lesions were present on the forelimbs and the ventral thorax, with thick crusts and scales (Fig. 1). Overall, the animal was in a good nutritional status and it was in lactation. The post-mortem examination did not show lesions compatible with myxomatosis or European brown hare syndrome.

**Fig. 2:**
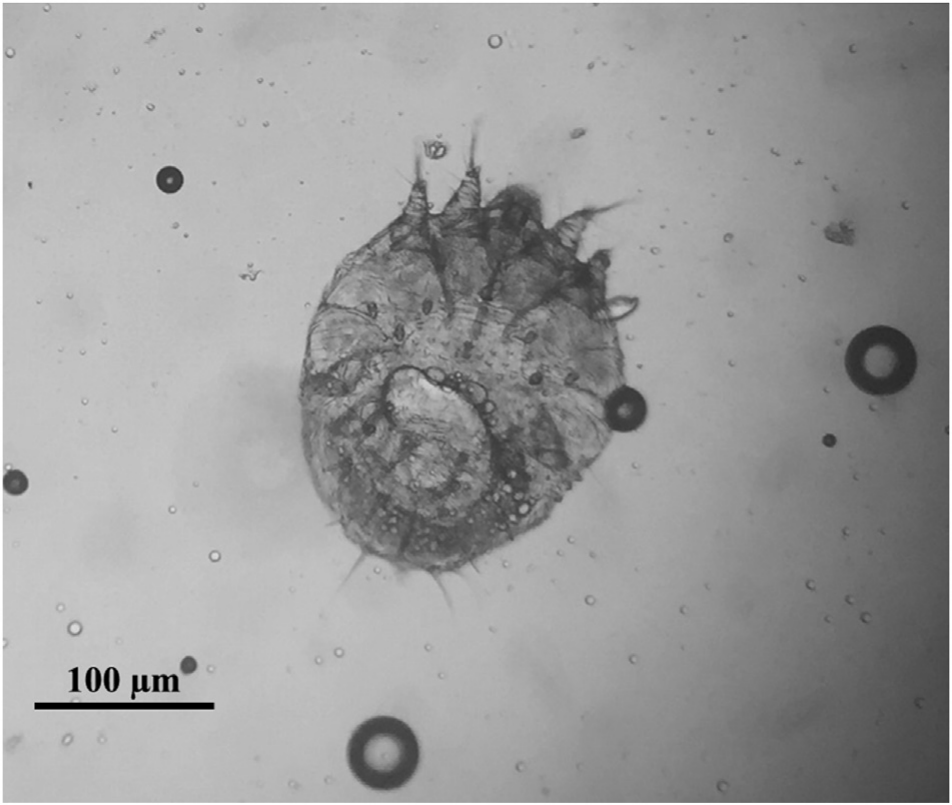
*Sarcoptes scabiei* mite collected from skin scrapings of the Iberian hare and identified under light microscope at a magnification of 100×

Skin samples were collected, and numerous mites of all developmental stages were microscopically observed. They were identified as *S. scabiei* (Fig. 2) based on morphological criteria (Mathison & Pritt, 2014). No other ectoparasites were observed on the animal.

Four mites from the hare were successfully isolated and processed for molecular analyses, whereas *S. scabies* mites previously obtained from other species and different populations were used as control groups (Table 1).

**Table 1:** Origin, sampling year and sample size of the animals affected by sarcoptic mange included in this study

Sampling site	Host species	*N*	*n*	Year
Valencia	*Lepus granatensis*	1	4	2019
Valencia	*Oryctolagus cuniculus*	1	3	2020
Catalonia (Tarragona)	*Oryctolagus cuniculus*	1	4	2010
Mallorca	*Oryctolagus cuniculus*	6	14	2010
Catalonia	*Vulpes vulpes*	8	20	2014
Valencia	*Vulpes vulpes*	2	3	2020

*Abbreviation*: *N*, number of sampled animals; *n*, number of mites used for Mst analysis.

A total of 56 alleles were detected. Allele count ranged from 3 (Sarms 34) to 10 (Sarms 33). Six private alleles were found across the 10 microsatellite loci, distributed among four loci (Sarms 33, 38, 41, 45). A significant deviation from HWE was detected throughout all loci, except for Sarms 34, 35, 37 (*P* < 0.05). Observed heterozygosity and allelic richness ranged between 0.07 (Sarms 37) and 0.25 (Sarms 35) and between 0.61 (Sarms 33) and 0.88 (Sarms 41), respectively (Table 2).

**Table 2:** Descriptive statistics of the *Sarcoptes* populations arranged by Sarms locus

Mst locus	He	Ho	R
Sarms 33	0.72	0.21	0.61
Sarms 34	0.63	0.09	0.82
Sarms 35	0.64	0.25	0.77
Sarms 36	0.57	0.07	0.81
Sarms 37	0.58	0.15	0.82
Sarms 38	0.66	0.20	0.70
Sarms 40	0.73	0.14	0.69
Sarms 41	0.28	0.11	0.88
Sarms 44	0.71	0.09	0.74
Sarms 45	0.71	0.13	0.67

*Abbreviations*: He, expected heterozygosity; Ho, observed heterozygosity; R, allelic richness.

**Fig. 3:**
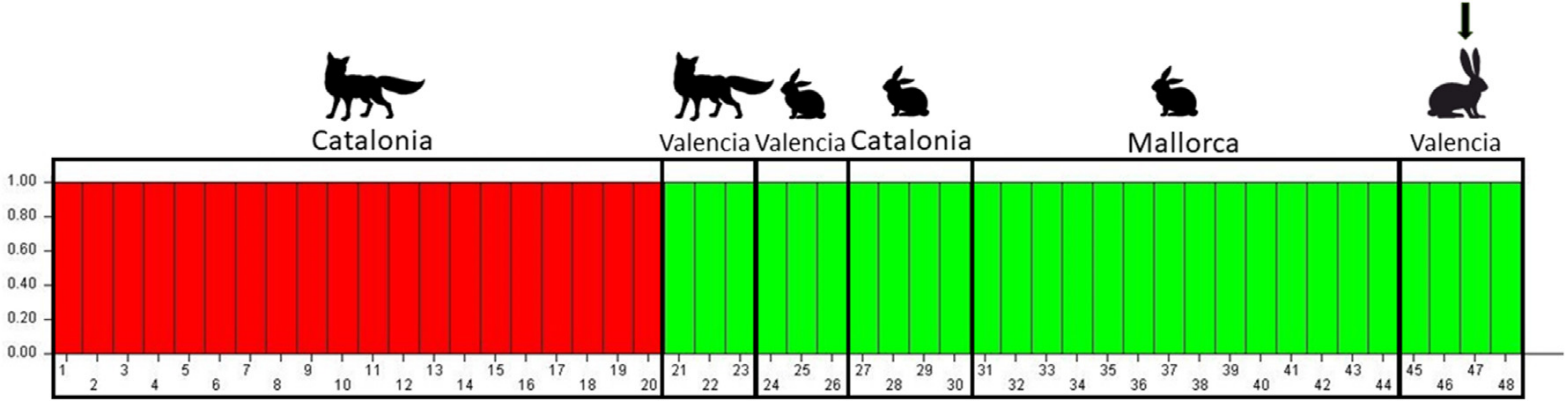
Bar chart of *Sarcoptes*-derived genetic cluster generated with the software Structure 2.3.4 with maximum likelihood *K* = 2. Each mite is represented by a single bar, and the height of each coloured segment is proportional to the membership fraction in each cluster. The arrow indicates the four mite samples from the Iberian hare of this study

According to the DK method of Evanno (*K* = 2), the Bayesian assignment test revealed the presence of two geographically separated *Sarcoptes*-derived clusters (Fig. 3), consisting of mites from the hare, wild rabbits and foxes from Valencia (green cluster), and mites from foxes originating from Catalonia (red cluster).

**Fig. 4:**
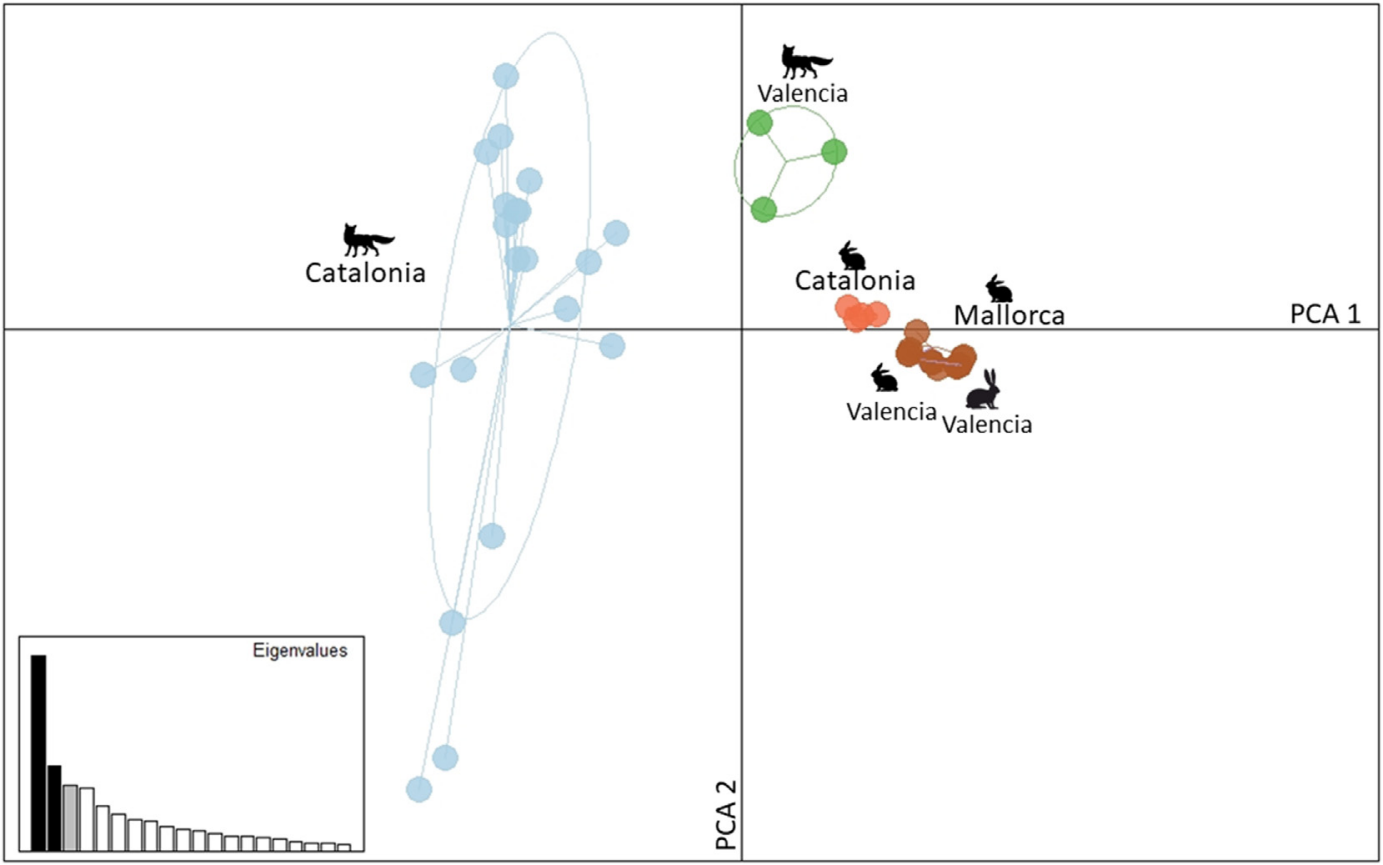
Principal components analysis (PCA) of microsatellite loci representing hare-, rabbit- and fox-derived mite populations in Spain. Each population is labelled with the host species and the geographical origin. Components 1 and 2 explained 14% and 6.1% of the variance, respectively (black bars of the eigenvalues). The eigenvalues of the two axes are displayed in the barplot on the left

The results of the PCA are displayed in Fig. 4. The multivariate analysis revealed three main clusters, prevalently scattered along the first axis: (i) mites collected on foxes from Catalonia; (ii) mites collected on foxes from Valencia; and (iii) mites collected on the hare from Valencia and wild rabbits from Catalonia, Valencia and Mallorca.

## 4 Discussion

In the past decade, microsatellite (Mst) markers have been widely used in *S. scabiei* epidemiological and population genetic investigations (Rasero et al., 2010; Gakuya et al., 2011; Matsuyama et al., 2019; Rudd et al., 2020; Moroni et al., 2021) and in forensic analyses (Alasaad et al., 2012). In the present study, ten Msts were used to molecularly type *Sarcoptes* mites derived from a free-ranging Iberian hare and trace the possible source of the infection.

According to the Bayesian assignment test (Fig. 3), the clustering of mites suggests that rabbits or sympatric foxes might be the actual source of infection for this hare, while the PCA analysis highlights three clusters, unambiguously showing that the hare-derived mites clustered with those from wild rabbits. This might be explained by the variance along the first axis of the PCA analysis (Fig. 4), contributing to a clear separation of fox-derived mites from Catalonia and the remaining mite samples, while the second axis separates the fox-derived mites from Valencia and the hare/rabbit-derived mites.

Descriptive genetic analysis revealed an evident deficiency in the observed heterozygosity and allelic richness, confirming the results of previous molecular investigations on *Sarcoptes* mite populations (Rasero et al., 2010; Gakuya et al., 2011; Matsuyama et al., 2019; Rudd et al., 2020; Moroni et al., 2021). Moreover, the low number of private alleles indicates a reduced genetic divergence and high gene flow between mite populations analyzed in this study.

Sarcoptic mange outbreaks were first reported in wild rabbits at the beginning of the 21st century, namely on Mallorca Island (Millán, 2010) and mainland southern Catalonia (Navarro-Gonzalez et al., 2010). However, results of a large-scale serosurvey showed that *S. scabiei* infection was endemic in wild European rabbits throughout the Iberian Peninsula since at least the 1990s of the previous century (Millán et al., 2012). Game restocking in the absence of effective sanitary control has been identified as a major risk factor for the spreading of sarcoptic mange amongst resident rabbits (Navarro-Gonzalez et al., 2010). On the other hand, the widespread exposure to *S. scabiei* infection in wild rabbits in Spain and the sympatry between wild rabbits and Iberian hares over large part of the respective distribution areas (Alves & Hackländer, 2008) suggest that *L. granatensis*, described here as a new host for *S. scabiei*, seems to be quite resistant to *S. scabiei* at both, individual and population level. This may be also true for other members of *Lepus* spp., in which sarcoptic mange has never been reported, to the best of our knowledge.

Skin lesions reported in this study resemble those observed in wild rabbits (Millán, 2010) and a single European brown hare (Restani et al., 1985), suggesting a similar clinical and pathological course of infection in susceptible individuals.

The clustering of mites collected on a fox from the Valencian Community with mites from wild rabbits and the Iberian hare (Fig. 3) is not surprising, since prey-to-predator transmission of *S. scabiei* has been already documented with molecular tools (Gakuya et al., 2011). However, the overall genetic results of this study (Bayesian assignment test and PCA analysis) confirm that gene flow between mites from similar host taxa occurs more frequently than between those from different sympatric host taxa, and thus, transmission of *S. scabiei* amongst individuals belonging to the same species or closely related ones, according to the “host-taxon law” (Rasero et al., 2010), is prevalent over other reported cross-transmission patterns. Moreover, it is worth noting that rabbit-derived *Sarcoptes* from Mallorca Island are in the same cluster of those originating from mainland southern Catalonia, despite the evident geographical limits (Fig. 3, Fig. 4).

The present report in a previously undetected host of *S. scabiei*, the Iberian hare, should raise awareness in wildlife operators and veterinary authorities, and stimulate monitoring programmes in areas where this endemic game animal shares range with endemically infected wild rabbits (Moroni et al., 2020). The role of restocking as a risk factor for the spread and persistence of sarcoptic mange in wild rabbits and sympatric lagomorphs should also be elucidated in the future.

## 5 Conclusions

We encourage further field and experimental investigations on possible *S. scabiei* cross-transmission between wild rabbits and the diverse representatives of *Lepus* spp. in Spain. The immunosuppressive role of selected viral agents should not be neglected, as adaptation of rabbit-derived *S. scabiei* to hares could be enhanced under such favourable circumstances. Current management practices (e.g. restocking of small game estates with farmed or wild-captured rabbits) should be also carefully assessed under this perspective.

## Funding

No funding was received for this study.

## Ethical approval

Not applicable.

## CRediT author statement

JC, LR, and BM designed the study. AMM coordinated the collection of the carcass and performed the post-mortem examination. SL and RV collected mite samples from Catalonia. BM performed the molecular analysis. JC drafted the first manuscript. JC, LR, VLL, SL, RV, BM and AMM finalized the manuscript. All authors read and approved the final manuscript.

## Data availability

Raw data used and analyzed during the present study are available from the first and the corresponding author upon reasonable request.

## Declaration of competing interests

The authors declare that they have no competing interests.

## Abbreviations

Sarms: *Sarcoptes* microsatellite
Mst: microsatellite
PCA: principal components analysis
